# A bi-ordering approach to linking gene expression with clinical annotations in gastric cancer

**DOI:** 10.1186/1471-2105-11-477

**Published:** 2010-09-23

**Authors:** Fan Shi, Christopher Leckie, Geoff MacIntyre, Izhak Haviv, Alex Boussioutas, Adam Kowalczyk

**Affiliations:** 1 National ICT Australia; 2 Department of Computer Science and Software Engineering, The University of Melbourne, Parkville, Victoria 3010, Australia; 3 Baker IDI Heart and Diabetes Institute, 250 Kooyong Road Caulield, Victoria 3162, Australia; 4 Peter MacCallum Cancer Center, St Andrew's Place, East Melbourne, Victoria 3002, Australia

## Abstract

**Background:**

In the study of cancer genomics, gene expression microarrays, which measure thousands of genes in a single assay, provide abundant information for the investigation of interesting genes or biological pathways. However, in order to analyze the large number of noisy measurements in microarrays, effective and efficient bioinformatics techniques are needed to identify the associations between genes and relevant phenotypes. Moreover, systematic tests are needed to validate the statistical and biological significance of those discoveries.

**Results:**

In this paper, we develop a robust and efficient method for exploratory analysis of microarray data, which produces a number of different orderings (rankings) of both genes and samples (reflecting correlation among those genes and samples). The core algorithm is closely related to biclustering, and so we first compare its performance with several existing biclustering algorithms on two real datasets - gastric cancer and lymphoma datasets. We then show on the gastric cancer data that the sample orderings generated by our method are highly statistically significant with respect to the histological classification of samples by using the Jonckheere trend test, while the gene modules are biologically significant with respect to biological processes (from the Gene Ontology). In particular, some of the gene modules associated with biclusters are closely linked to gastric cancer tumorigenesis reported in previous literature, while others are potentially novel discoveries.

**Conclusion:**

In conclusion, we have developed an effective and efficient method, Bi-Ordering Analysis, to detect informative patterns in gene expression microarrays by ranking genes and samples. In addition, a number of evaluation metrics were applied to assess both the statistical and biological significance of the resulting bi-orderings. The methodology was validated on gastric cancer and lymphoma datasets.

## 1 Background

A typical aim of exploratory analysis of genomics data is to identify potentially interesting genes and pathways that warrant further investigation. There is a critical need to streamline the analysis in order to support continuing advances in high throughput genomics methods such as gene expression microarrays, which measure thousands of genes in a single assay and are the focus of this paper. However, such assays provide noisy and incomplete measurements, which require sophisticated bioinformatics techniques to identify statistically and biologically significant associations between genes and relevant phenotypes of interest.

Unsupervised analysis techniques cluster data without using prior information on the labels of samples. This enables the discovery of novel histological subtypes. However, a major limitation of traditional clustering algorithms for this task is that they cluster either genes or samples into non-overlapping groups, based on the similarity of gene expression across all samples for gene clustering, or all genes per sample in sample clustering. This limits the ability to find groups of genes that are "co-correlated" across only a *subset *of samples, or participate in multiple cellular pathways. A related open issue is how to evaluate the statistical significance of the clusters.

In spite of such limitations, there are examples of remarkable biologically significant discoveries. One such case revisited in this paper is the analysis of gastric cancer data [1]. The original paper used hierarchical clustering of both 7383 genes and 124 gastric cancer samples (malignant and pre-malignant). By inspecting the "heat map" they observed a number of prominent biclusters, which were linked to various aspects of cancer etiology. However, the approach [1] was heavily dependent on manual inspection to identify the groupings. In particular, several sets of co-expressed genes were not grouped together by hierarchical clustering, and needed to be grouped manually by expert analysis. Moreover, it is difficult to assess whether such clusters are robust to any changes, and whether different clustering attempts converge to a stable result. Consequently, there is a need for techniques that can guide such a process of discovering significant and worthwhile hypotheses for follow-up analysis.

Biclustering, also called co-clustering, is a promising technique proposed for the automated discovery of highly correlated subsets of genes across a subset of samples. The concept of "biclustering" was first introduced by [2] and has been the subject of several surveys [3,4]. Many techniques have been used for finding biclusters with different objective functions, such as "SAMBA" using graphic models [5], biclustering by Gibbs sampling [6], the Order-Preserving Submatrix algorithm (OPSM [7]), biclustering using maximum-similarity between genes [8], the Iterative Signature Algorithm (ISA [9]), and biclustering using linear geometry [10]. Recently, several studies have applied biclustering to more specific bioinformatics areas, such as local multiple sequence alignment of RNA [11] and e-CCC-Biclustering for gene expression time-series data [12]. Several of these representative biclustering methods will be used as a basis for comparison in this paper.

This paper proposes a technique for exploratory biclustering analysis, which combines biclustering with an evaluation of the statistical significance and biological relevance of such biclusters. There are four main contributions that we make in this paper. First, we introduce a novel algorithm, called *bi-ordering*, which is in some respects a member of the family of biclustering techniques. This algorithm is benchmarked against several relevant biclustering algorithms in the literature [2,5,6,9]. Second, we extend an existing statistic based on the hyper-geometric distribution to a generalized statistic for evaluating the saturation of phenotypes in biclusters, called the Multiple-Class-Saturation (MCS) metric. In addition, we apply the Jonckheere trend test [13] to evaluate the significance of the correlation between ordered samples and clinical annotations. Third, we assess the stability of the observed results by assessing the size of their "basin of attraction" as follows. In our experiments, random initializations of the algorithm yield many unique biclusters, which are then grouped into a manageable number of families of very similar outcomes (called a "super-bicluster") by a secondary clustering of the biclusters. The size of these super-biclusters provides a measure of bicluster "stability". We find that our technique is able to find a small set of highly stable super-biclusters, which correspond to distinct histopathological types in an existing gastric cancer dataset [1]. We have also applied our approach to analyze a lymphoma dataset [14]. Fourth, we demonstrate that the discovered super-biclusters have associated Gene Ontology (GO) terms with very significant p-values, which can provide a basis for the biological interpretation of the gene modules.

In Section 2, we introduce our core algorithm BOA, as well as the statistics for its evaluation. In Section 3, we validate the BOA algorithm on the gastric cancer and lymphoma datasets and compare the results to other algorithms. The biological interpretations of the results from the gastric cancer datasets are discussed in Section 4, 5 and the Additional file 1. Finally, we conclude the paper in Section 6.

## 2 Methods

In this section, we introduce a protocol for identifying and characterizing modules of genes and orderings of samples that exhibit high statistical, biological and clinical significance. Our protocol, named Bi-ordering Exploratory Analysis (BEA), comprises six main stages as described below.

### Bi-ordering Exploratory Analysis

Input: A gene expression data matrix [*x_gs_*]*n_G _*× *n_S _*for *n_G _*genes for *n_S _*samples.

1. Generate biclusters with ordered genes and samples using BOA algorithm outlined below.

2. Merge similar biclusters into "super-biclusters" to identify robust modules of co-expressed genes.

3. Annotate biclusters with histological and biological attributes to support their interpretation.

4. Generate figures of merit (i.e., p-values) for each bicluster:

(a) Over-representation of histological categories;

(b) Concordance of sample orderings with various phenotype gradients;

(c) Gene Ontology annotations.

5. Evaluate biological significance of results.

Now we briefly elaborate on selected key stages of the above protocol.

### 2.1 Bi-ordering Analysis

In this section, we describe Bi-ordering Analysis and its details.

#### 2.1.1 Algorithm

The term "biclustering", introduced by Cheng and Church [2] refers to the identification of a sub-matrix with "significantly homogeneous entries". In this section we introduce a novel algorithm called Bi-ordering Analysis (BOA), which is pivotal in the generation of our results. We now introduce our BOA algorithm. For the sake of clarity, we define an averaging operation as follows. For a vector *s_i _*and a set of indices *I *with finite cardinality (0 < |*I*| < + ∞) we introduce the following notation for the average:

$${\langle s_{i}\rangle }_{i\in I}=\sum_{i\in I}s_{i}/|I|.$$

**Algorithm 1 ***Bi-Ordering Analysis (BOA)*

Input: *n_G _*× *n_S _*gene expression data matrix [*x_gs_*]; two cut-off thresholds *θ_G _*and *θ_S _*for selecting genes and samples, respectively.

1. Normalize data: first, for each gene across all samples, normalize to *median *= 0 and *std *= 1, then repeat this for each sample across all genes.

2. Initialization: Select a subset of samples ∅ ≠ *S *⊂ {1, ⋯, *n_S_*}

3. Repeat the Steps a-d below until convergence (i.e., *G *and *S *stabilise):

(a) Update gene scores *f*(*g*) ← ⟨*x_gs_*⟩_*s*∈*S *_for *g *= 1, ⋯, *n_G_*,

(b) Select genes: $\text{G}\leftarrow \{g;f(g)-{\langle f(g)\rangle }_{g=1,\cdots ,n_{G}}>\theta_{G}/\sqrt{\left|S\right|}\}$,

(c) Update sample scores *h*(*s*) ← ⟨*x_gs_*⟩_*g*∈*G *_for *S *= 1, ⋯, *n_S_*,

(d) Select samples: $S\leftarrow \{s;h(s)-{\langle h(s)\rangle }_{s=1,\cdots ,n_{S}}>\theta_{S}/\sqrt{\left|G\right|}\}$,

Output: The gene scores *f*(*g*), sample scores *h*(*s*), and a bicluster *B *= (*G*, *S*), which is determined by applying the cut-off thresholds *θ_G _*and *θ_S _*to the scores *f*(*g*) and *h*(*s*), respectively.

According to the iterative process of step 3 in the BOA algorithm, we aim to select a submatrix with coherently high gene expression levels across selected samples. In particular, we aim to detect differentially expressed genes between phenotypes. Consequently, it is important to normalize the data in step 1, otherwise, the genes with high expression levels across most samples will dominate the selection procedure. The above algorithm is similar to ISA [9], which is discussed in more detail in Section 3.2.

#### 2.1.2 Variants of BOA

Note that two further variants of BOA are possible:

1. **Under-expressed biclusters **- This variant finds significantly down-regulated genes by selecting genes and samples with *f*(*g*) and *h*(*s*) less than a threshold in steps 3.b and 3.d, respectively.

2. **Fixed-size biclusters **- We could also select *G *and *S *of fixed size rather than using the cut-off thresholds in each iteration. The attraction is that the algorithm is guaranteed to converge, which in practice may not always hold for the previous two options. The formal proof of the convergence for this variant is included in the Additional file 1.

#### 2.1.3 Ordering scores

An important ability of the BOA algorithm is to assign an ordering score *h*(*s*) to samples and *f*(*g*) to genes in a bicluster. The sample score *h*(*s*) orders all samples according to the average expression level across a subset of genes in the current bicluster, while the gene score *f*(*g*) orders all genes according to a subset of samples. The sample score *h*(*s*) relates a bicluster to the clinical annotations of samples, especially for multi-class attributes, such as the cancer progression stage, or continuous attributes, such as the survival time. These are discussed in detail in Section 2.3.

### 2.2 Super-biclustering (SBC)

Super-biclustering is an important feature of our technique, in terms of improving the robustness and stability of the results. For every individual bicluster, the ultimate output of gene score *f*(*g*) and sample score *h*(*s*) are uniquely determined by the selection of the initial subset of samples *S*. This is because every selection in Step 3 of Algorithm 1 is deterministic for a given pair of thresholds (*θ_G_*, *θ_S_*). Thus, different initializations may result in different biclusters, which are local optima after a few iterations. In order to cover more potential biclusters, we run BOA with 1000 different initial subsets of samples. In every independent initialization, each sample is drawn into the initial subset randomly with probability of 0.2. Some of the 1000 generated biclusters are identical, while others may be very similar to each other but not exactly the same due to local optima. In order to identify robust and distinct biclusters, we then apply a hierarchical clustering algorithm to group similar biclusters into *super-biclusters (SBC)*. We use the Jaccard coefficient on gene sets as a similarity measure between two biclusters. For example, given two biclusters *B*_1 _= (*G*_1_, *S*_1_) and *B*_2 _= (*G*_2_, *S*_2_), the similarity between them is:

(1) $$sim(B_{1},B_{2})=\frac{\left|G_{1}\cap G_{2}\right|}{\left|G_{1}\cup G_{2}\right|}$$

The similarity measure could also be defined on samples, though here we focus on genes, which are the dominant and far more complex dimension to handle in gene expression microarray datasets. Based on the similarity measure in Equation 1, a hierarchy of unique biclusters is constructed. We then use a similarity threshold of 0.5 to extract a few top groups of biclusters, which are super-biclusters, from this hierarchy. For convenience of evaluation, a typical bicluster is selected as a prototype in each resulting SBC. Without loss of generality, we choose the bicluster that covers the most genes in its SBC as the prototype, and so any further numerical evaluation is based on these prototypes when we refer to a SBC.

### 2.3 Figures of merit

In order to evaluate the statistical and biological significance of the resulting biclusters, we employ three statistical methods and their corresponding p-values.

#### 2.3.1 Saturation metric of samples

The homogeneity of phenotypes of samples in a bicluster can be evaluated if we are given a prior classification of each sample (e.g., its cancer subtype) as the label. Ideally, each bicluster should be enriched with samples in one or a few more similar classes, e.g., normal or tumor samples. For the purpose of quantification, we use the p-value of the hypergeometric distribution to evaluate the purity of biclusters according to the phenotypes of samples. Previously [5], a measure of homogeneity using the hypergeometric distribution was applied to the single most abundant class within a bicluster. However, if some genes are co-expressed across multiple classes, calculating p-values on a single class is not an adequate representation of accuracy. To address this limitation, we extend this measure to a more generalized form where the significance is calculated for a group of classes to determine the dominant class(es). We refer to the original statistic used in [5] and our generalized statistic as Single-Class Saturation (*SCS*) and Multiple-Class Saturation (*MCS*) metrics, respectively. The calculation of MCS p-values based on the hypergeometric distribution is given in Equation 2 below.

Given a classification of samples with *q *classes {*C*_1_, ⋯, *C_q_*} and a bicluster *B *= (*G*, *S*), the p-value with respect to a group of *r *classes $\left\{C_{i_{1}},\cdots ,C_{i_{r}}\right\}$ is computed by:

(2) $$p_{MCS}(B)=\sum_{x=k}^{\min(m,|S|)}\frac{\left(\begin{array}{c} m\\ x \end{array}\right)\left(\begin{array}{c} n_{S}-m\\ |S|-x \end{array}\right)}{\left(\begin{array}{c} n_{S}\\ \left|S\right| \end{array}\right)}$$

where

$$\begin{array}{l} m:=\left|\underset{C=C_{i_{1}},\cdots ,C_{i_{r}}}{\cup }\left\{s;class(s)=C\right\}\right|,\\ k:=\left|\underset{C=C_{i_{1}},\cdots ,C_{i_{r}}}{\cup }\left\{s\in S;class(s)=C\right\}\right| \end{array}$$

are the numbers of samples in the dataset and in the bicluster *B *annotated with any class in $\left\{C_{i_{1}},\cdots ,C_{i_{r}}\right\}$, respectively. This quantity calculates the probability of observing *k *or more samples classified in $\left\{C_{i_{1}},\cdots ,C_{i_{r}}\right\}$ in a bicluster *B*.

In our evaluation, we generate the full set of combinations of all sample classes from {*C*_1_, ⋯, *C_q_*} and compute *p_MCS _*for each bicluster and each combination, so that we could discover any potential associations between gene sets and a group of phenotypes. Finally, we select the subset of classes that corresponds to the most significant p-value for each bicluster in the evaluation in Section 3. Note that the SCS is a special case of the MCS. We compute a p-value with respect to each individual class, and then select the single class that corresponds to the best p-value for each bicluster.

#### 2.3.2 Jonckheere's trend test

Another method to evaluate the significance of a bicluster is to compare the ordering of all samples *h*(*s*) generated by BOA with any relevant ordering *y*(*s*) of all samples based on their biological annotations, e.g., the progression stage of the cancer in the sample. We can test the agreement of samples ordered according to *h*(*s*) with this progression score *y*(*s*). We use Jonckheere's test statistic [13]:

(3) $$U=\left|\left\{\left(s,s^{'}\right);h\left(s\right)<h\left(s^{'}\right)and\;y\left(s\right)<y\left(s^{'}\right)\right\}\right|$$

for this purpose.

For a random scoring *h*(*s*) (the null hypothesis), this random variable *U *has an approximately normal distribution. For example, consider that we have an annotation scoring *y*(*s*) of samples with respect to *q *sample classes {*C*_1_, ⋯, *C_q_*}, which can be numerically ranked, e.g.,

(4) $$y\left(\left\{s|s\in C_{1}\right\}\right)<y\left(\left\{s|s\in C_{2}\right\}\right)<\cdots <y\left(\left\{s|s\in C_{q}\right\}\right)$$

Let *N_i _*(1 ≤ *i *≤ *q*) denote the number of samples in class *C_i_*, and *N *denote the total number of samples. The approximate normal distribution of *U *determined by the random scoring *h*(*s*) and the annotation scoring *y*(*s*) has the mean:

(5) $$\sum_{1\leq i<j\leq q}N_{i}N_{j}/2$$

and the variance

(6) $$[N^{2}(2N+3)-\sum_{1\leq i\leq q}N_{i}^{2}(2N_{i}+3)]/72$$

from which the p-values can be estimated.

#### 2.3.3 Gene Ontology Annotations

Given that each gene's expression in a bicluster is highly similar with respect to other genes in the bicluster, it is expected that the collection of genes as a whole are likely to be involved in some related biological processes. In order to determine this, the structured vocabulary of the Gene Ontology (GO) [15] is used to help uncover the biological processes represented by each of the biclusters. As each gene can be annotated with one or more terms within the GO, we can determine which GO terms are statistically over-represented within a group of genes. We use an existing tool GOstat [16] to determine the statistically over-represented terms within each bicluster for the biological process branch of the GO.

### 2.4 Efficiency

One of the advantages of the BOA algorithm is its efficiency. The time complexity in each iteration is O(*n_G _*+ *n_S_*), since only averaging operations for computing the gene score *f*(*g*) and sample score *h*(*s*) are required. Practically, the number of iterations for generating a single bicluster is usually no more than 10, and the number of initializations is 1000 in our experiments.

## 3 Results

In this section, we analyze the performance of our algorithm on a real gene expression dataset, namely the gastric cancer dataset in [1]. The main reason for this choice is the availability of local expertise in the biology of this disease. We compare the performance of our algorithm in terms of SCS and MCS in Section 2.3 to the results obtained from the algorithms in [2,5,6,9] by using the parameter settings recommended in those papers, including the normalization method specified in each algorithm, or by observing the best results obtained under different parameter settings. The evaluation using Jonckheere's test, the Gene Ontology and the biological relevance of the results for gastric cancer are discussed in detail in Section 4. In addition, we also apply BOA to another lymphoma dataset for validation [14].

### 3.1 Results of BOA on Gastric Cancer dataset

After applying gene filtering as described in [1], we have *n_G _*= 7383 gene expressions evaluated for *n_S _*= 124 human tissue samples. Excluding two singletons, there are six different phenotypes in the data, of which three are subtypes of gastric cancer: 35 diffuse (DGC), 22 intestinal (IGC), 7 mixed (MGC); and the other three phenotypes are pre-malignant conditions: 26 chronic gastritis (CG), 22 intestinal metaplasia (IM) and 10 normal, e.g., non-inflamed mucosa tissue removed during surgery for the gastric cancer. Now we briefly discuss the algorithmic aspects and setup of the experiment.

First, we generated a set of 1000 initializations, which were 1000 subsets of samples selected by the method described in Section 2.2. The actual number of initializing samples for gastric cancer data ranged from 14 to 41 across 1000 subsets. As described in Section 2.2, each sample is randomly selected with a probability of 0.2 for inclusion in the initial subset of samples. Note that other selection probabilities of 0.4, 0.6 and 0.8 have been tested, but the results were largely insensitive to changes in this parameter.

Note that in the BOA algorithm, there are other alternative normalization methods that can be used, i.e., using *mean *= 0 instead of *median *= 0 for centering the genes and samples. Here, we followed the normalization method used in [1] for the sake of a fair comparison with their manual analysis. In addition, we have found that there is very little numerical difference between normalizing by *median *= 0 and normalizing by *mean *= 0 on the dataset we have studied.

Second, we applied BOA to the gastric cancer data using 11 different pairs of thresholds: (*θ*_G_, *θ_S_*) = (5.0, 3.0), (4.0, 4.0), (5.0, 4.0), (5.5, 4.0), (6.0, 4.0), (5.0, 4.5), (5.5, 4.5), (5.0, 5.0), (5.5, 5.0), (6.0, 5.0), (5.5, 5.5), which used the same set of initializations.

These threshold settings were limited to this range since they produced biclusters of moderate size. For all biclusters across the 11 pairs, the minimum and maximum number of genes were 21 and 816, respectively. We have also tried several other groups of thresholds on the dataset, but the generated biclusters are not very informative if the thresholds are too large or too small.

For the resulting biclusters with each setting, we found that the minimal p-values ranged between 4.3 × 10^-10 ^and 1.6 × 10^-9 ^for the SCS metric (no major difference was observed for SCS with 9 of the 11 threshold settings achieving the minimum p-value of 4.3 × 10^-10^), and between 3.4 × 10^-27 ^and 4.0 × 10^-14 ^for the MCS metric. For further analysis we chose a mid-range pair *θ_G _*= 5 and *θ_S _*= 4.5 for which, additionally, all 1000 initializations of BOA converged. Under this pair of thresholds, the algorithm converged to 49 biclusters, which were further grouped into 8 super-biclusters (see Table 1), and a prototype bicluster was chosen for each super-bicluster as described in Section 2.2.

**Table 1:** Super-biclusters in gastric cancer dataset

	#Converging			P-values		Most significant biological process
	
SBC	SBC	Prototype	MCS	Malignancy Score	GO	
SBC1	11	6	9.4 × 10^-4^	1.8 × 10^-13^	5.1 × 10^-9^	epidermis development

SBC2	188	7	1.0 × 10^-8^		7.1 × 10^-7^	lipid metabolic process

SBC3	2	1	1.5 × 10^-6^	5.5 × 10^-8^	3.2 × 10^-32^	immune system process

SBC4	96	2	1.8 × 10^-1^		2.0 × 10^-53^	immune system process

SBC5	15	15	1.1 × 10^-18^	7.7 × 10^-21^	1.8 × 10^-14^	cell cycle process

SBC6	328	11	3.0 × 10^-7^	4.9 × 10^-8^	1.8 × 10^-20^	multicellular organismal process

SBC7	359	229	4.0 × 10^-14^	-5.4 × 10^-22^	3.2 × 10^-22^	gen. of precursor metabolic & energy

SBC8	1	1	3.0 × 10^-10^	-5.2 × 10^-8^	2.2 × 10^-2^	lipid metabolic process

Numerical characterisations and biological relevance of the eight super-biclusters generated by BOA on the gastric cancer data. In the second column of the table, the numbers of biclusters that converged to a particular super-bicluster are given, while the third column is the number of identical biclusters converging to the prototype of that super-bicluster. The columns of "MCS", "Malignancy Score" and "GO" contain the p-values calculated with respect to the prototype of each super-bicluster in terms of the three statistics described in Section 2.3. Note that the negative sign, '-', in the Malignancy Score for SBC7 and SBC8 indicates the significance of agreement with the reverse order.

**Figure 1:**
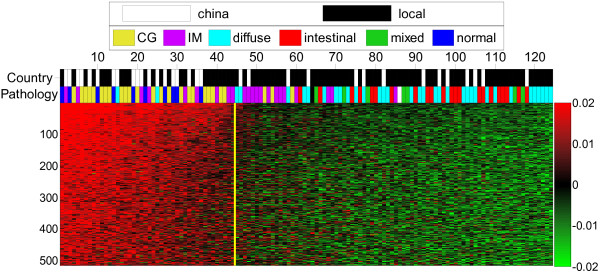
**Heat map of super-bicluster 7**. Heat map for the prototype of the most prominent super-bicluster, SBC7, generated by the BOA algorithm for the gastric cancer data. The vertical axis shows the 515 most significant genes ordered by *f*(*g*) in Algorithm 1, and cut by *θ_G _*= 5.0, while the horizontal axis shows all samples ordered by *h*(*s*) and cut by *θ_S _*= 4.5. The yellow vertical line in the middle of figure indicates the boundary between the samples in the bicluster (left-side) and others (right-side). The bicluster samples are enriched with the CG subtype with a p-value of 4.32 × 10^-10 ^in terms of the SCS metric or enriched with a combination of {normal, CG, IM} subtypes with a p-value of 4.03 × 10^-14 ^in terms of the MCS metric. Moreover, we observe a strong gradation from least malignant samples (normal and CG), through an intermediate phenotype IM, to the malignant samples (combined intestinal, diffuse and mixed gastric cancers). Two phenotypes, squamous and adenosquamous, with only one sample are annotated with black and white, respectively, but are not shown on the legend. The probability of obtaining such or better ordering by random chance was estimated to have a p-value of 5.35 × 10^-22 ^in terms of Jonckheere's test.

To show the significance of the resulting biclusters we focus on the most stable super-bicluster generated for the gastric data, labeled SBC7 in Table 1. Its prototype is shown in Figure 1. The BOA algorithm converged to this super-bicluster 359 times out of 1000 initializations and its prototype 229 times out of 1000. This bicluster contains 515 genes that are prominently over-expressed in most pre-malignant samples and are under-expressed in most malignant samples. Numerically, it results in *p_SCS _*= 4.32 × 10^-10 ^with respect to the SCS metric (dominant class is CG) and a *p_MCS _*= 4.03 × 10^-14 ^with respect to the MCS metric (dominant classes are Normal, CG and IM).

However, there are two limitations of calculating SCS or MCS. First, these measures cannot deal with the case of continuous annotations of samples. Second, the significance of SCS and MCS are affected by the choice of cut-off threshold on samples, especially when the sample orderings *h*(*s*) change smoothly. Thus, we also used Jonckheere's test to overcome these limitations. We first allocated a "Malignancy Score" *y*(*s*) to each sample s following the expert advice: *y*(*s*) = 1 for normal, 2 for CG, 3 for IM and finally 4 for any gastric cancer (DGC, IGC or MGC sample). We then tested the significance of the agreement of the samples ordered according to the *h*(*s*) score generated by the BOA algorithm with this progression *y*(*s*). For the prototype of SBC7, the malignancy scores show an increasing trend from normal (*y*(*s*) = 1) to malignant samples (*y*(*s*) = 4) along the ascending ordered gene expression levels, which results in a directional p-value of 5.35 × 10^-22^.

For every bicluster, we used the GOstat program [16] to obtain significantly over-represented GO terms to investigate the associations between the terms and phenotypes. The GOstat program assesses the enrichment of GO terms within a group of genes by computing p-values from the *χ*^2 ^distribution. The p-values were corrected by the process of controlling the False Discovery Rate [17] in our experiment. As an example, several of the most significant GO terms of SBC7 are shown in Table 2.

**Table 2:** Over-represented GO terms in gastric cancer dataset

ID	P-value	Biological process
GO:0006091	3.24 × 10^-22^	generation of precursor metabolites and energy

GO:0006119	3.68 × 10^-18^	oxidative phosphorylation

GO:0006118	3.48 × 10^-12^	electron transport

GO:0042773	8.12 × 10^-12^	oxidative phosphorylation#ATP synthesis coupled electron transport

GO:0042775	8.12 × 10^-12^	organelle ATP synthesis coupled electron transport

The five most significantly over-represented GO terms associated with the genes of the prototype of SBC7.

The results are generated from GOstat [16].

More biological details of the gene modules and evaluation statistics for different SBCs are discussed in the next section.

### 3.2 Comparison with other algorithms

**Figure 2:**
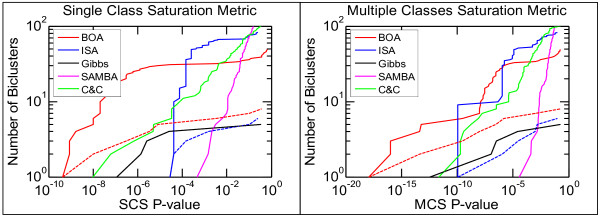
**Saturation metrics for gastric cancer dataset**. Gastric cancer benchmark results for five biclustering algorithms. We plot the number of unique biclusters (solid lines) and super-biclusters (broken lines) with the p-value below the threshold indicated by the x-axis. Each algorithm is represented with a unique color as shown in the legend. The results for the super-biclusters are represented with the same color as the biclusters for BOA, ISA and Gibbs (broken lines). Note that Gibbs produces exactly the same lines for biclusters and super-biclusters due to their algorithm. We have used the SCS (left sub- figure) and MCS (right sub- figure) metrics to calculate the p-values. We have applied 1000 random initializations for BOA and ISA and the parameter settings follow the suggestions in these studies.

As a basis for comparison with our BOA algorithm, we have also tested several existing biclustering algorithms, namely, Cheng and Church's algorithm [2], SAMBA [5], biclustering by Gibbs sampling [6] and the ISA algorithm [9], which is closest to our algorithm. We have used publicly available implementations of these algorithms in our evaluation, i.e., SAMBA is tested using Expander [18], Gibbs sampling has been implemented by ourselves, and the biclustering toolbox BicAT [19] is used for the other two algorithms. We compare the results of BOA with four other algorithms in terms of the SCS and MCS metrics as shown in Figure 2. In these figures, we plot the number of biclusters (vertical axis) whose SCS or MCS p-values are less than a given value on the horizontal axis, indicating the ability of each algorithm to find significant biclusters in terms of the SCS and MCS metrics. However, most algorithms usually generate redundant biclusters to different extents, i.e., slightly different biclusters due to local optima, and so comparing the redundant biclusters is not a fair evaluation. To eliminate the impact of redundancy, we applied the same super-biclustering process described in Section 2.2 to the resulting biclusters in each algorithm, and the results are shown as the lines with circle markers in the same figure. Note that the Gibbs algorithm [6] has the same biclusters and super-biclusters due to their strategy. SAMBA and Cheng & Church's algorithm still yield a large number of insignificant super-biclusters so they are not included. Generally, the BOA algorithm produced the most significant biclusters in terms of the p-values of both the SCS and MCS metrics.

Cheng & Church's method was chosen as a comparison because it is similar to our BOA method in two aspects. First, they both aim to identify "constant biclusters" [3]. Second, they all use an iterative process to refine a bicluster. However, the BOA algorithm has the following advantages that differ from Cheng & Church's method. First, in every step of the iterative process, BOA ranks all the genes and samples based on the current subset of samples and genes, so that the converged biclusters are local optima solutions that imply the relevance between genes and samples. The relation between genes and samples is a desirable property in biology, especially when studying the continuous annotations of samples. Second, the super-biclustering is also an important part of our framework, which identifies similar biclusters and then creates representative prototypes of biclusters, so that more meaningful biclusters could be found. In addition, to make a fair comparison, we also applied Cheng & Church's method to the gastric cancer dataset that has been normalized by the procedure described in the BOA method. The resulting biclusters of the two methods were evaluated by the saturation metrics and reported in the Additional file 1.

The BOA algorithm is very similar to ISA. However, the main objective of ISA is discerning "co-regulated" gene modules, while the association with phenotype classes (conditions) is not important, whereas it is of prime interest for our medical application. The main formal differences resulting in the different performance are: (i) ISA starts with an initialisation of a subset of genes; (ii) the two sided test is used for the selection of samples; (iii) samples are weighted, with possibly negative weights, so different conditions, say with up-regulated and down-regulated genes, can be joined in the same bicluster. Consequently, ISA aims at generating "constant column" biclusters while BOA's objective is a "constant" bicluster [3].

Figure 2 shows that BOA generates more significant biclusters in terms of SCS and MCS.

Our evaluation of GO annotations for both ISA [9] and biclustering by Gibbs sampling [6] show that they are capable of generating biclusters of significance comparable to BOA (details of values are not shown). These algorithms generated 6 and 5 SBCs, respectively, with similar gene sets to the SBCs of BOA. For example, the GO annotations "generation of precursor metabolites and energy" and "oxidative phosphorylation" significantly associated with SBC7 of BOA whose p-values are 3 × 10^-22 ^and 4 × 10^-18 ^(in Table 1) are also found by the ISA algorithm with p-values of 3 × 10^-8 ^and 4 × 10^-6 ^and Gibbs algorithm with p-values 1 × 10^-30 ^and 5 × 10^-13^. Similarly, the "multicellular organismal process" and "multicellular organismal development" annotations (significant for diffuse-type gastric cancer) in SBC6 of BOA, were also found by the ISA and Gibbs algorithms. However, we have observed that the BOA algorithm usually has better performance than either ISA or Gibbs in terms of Jonckheere's test, in particular, in the case of the evaluation of the "Malignancy Score".

### 3.3 Validation on lymphoma dataset

**Figure 3:**
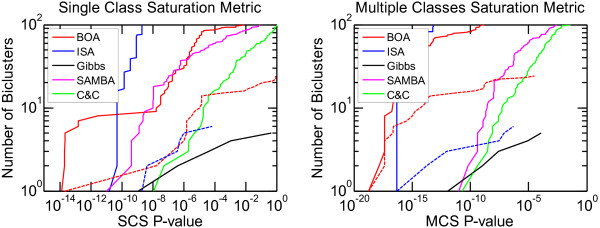
**Saturation metrics for lymphoma dataset**. Lymphoma dataset benchmark results for five biclustering algorithms. The experimental settings and elements of these figures are the same as the gastric cancer experiments.

To further validate the performance in terms of SCS and MCS, we applied BOA to a lymphoma dataset [14], and compared the result to the benchmark results of the other four algorithms. Similar figures of the SCS and MCS p-values are drawn and show in the Additional file 1. Again, the BOA algorithm generated very significant results in terms of identifying pathological categories (See Figure 3 for details).

## 4 Biological Analysis of Gastric Cancer

In this section, we focus on validating the biological significance of our findings for the gastric cancer dataset.

### 4.1 Gene modules compared with previous study

We first compare the gene modules of the prototypes of the super-biclusters with those reported in a previous study [1]. In that study, hierarchical clustering was applied to the gastric cancer dataset (cDNA platform) and several regions of genes related to different cancer types or pre-malignant states were annotated (labeled A - K in Figures 1 &2[1]. To validate the biological functions of our biclusters, we determined the intersection between the genes in these identified regions and the genes appearing in the prototypes of the eight super-biclusters (SBC1 - SBC8) discussed in Section 3.1. The results are shown in Table 3. Note that the two largest super-biclusters (SBC6 and SBC7) were a close match for the two most prominent gene clusters annotated as regions B & K [1]. Moreover, the super-bicluster SBC2 linked two separated but related biclusters in regions E & F [1], while the regions D1 to D3 that needed to be manually grouped in the hierarchical clustering [1] were automatically grouped by our method in SBC5. These unique biclusters confirm the homogeneous functions of the disjoint gene sets generated by hierarchical clustering.

**Table 3:** Comparison with previous literature

	**Region in **[1]		SBC1	SBC2	SBC3	SBC4	SBC5	SBC6	SBC7	SBC8
Symbol	Annotation	No. Genes	41	217	194	158	227	409	515	146

B	Mitochondrial	665	0	0	0	0	0	1	416	9

D1-D3	Proliferation	201	0	0	0	0	76	0	0	0

E	Intestinal	294	1	81	0	0	0	0	1	44

F	Intestinal	157	0	112	0	0	7	1	0	27

G	Squamous	37	25	0	0	0	0	0	0	0

H	Inflamation	330	7	0	117	135	9	7	0	30

K	Extra cellular matrix	877	3	0	67	0	74	392	1	0

Overlapping genes between prototypes of super-biclusters and functional regions in [1]. In the second row we show the number of genes in the SBC prototype.

### 4.2 Biological relevance for gastric cancer

In Table 3 we then considered the significance of these super-biclusters in terms of the three types of figures of merit discussed in Section 2.3, namely, the SCS and MSC p-values, the p-value of the over-represented GO annotations, and the p-value of the Jonckheere test on the order of the progression of the cancer in the samples. We have discussed the assignment of malignancy scores *y*(*s*) and tested the significance of the agreement between *y*(*s*) and sample orderings *h*(*s*) in Section 3.1. Table 1 shows the numerical results of these statistics. The heat map of SBC7 (Figure 1) shows that the ordering induced by the bicluster has a clear negative correlation with the malignancy score of the samples. The *h*(*s*) for SBC5 and SBC7 and to a lesser extent SBC3 are very significantly correlated with *y*(*s*). More biological relevance is discussed in the Discussion section.

## 5 Discussion

Based on the results of our experiments, we now consider the biological significance of our findings. The generated results including the GO and clinical correlation were analysed by expert biologists and clinicians. We quote them to some extent as a proof that the formal data processing protocols as discussed here can lead to the generation of significant biological hypotheses warranting follow-up wet lab experiments. The BOA algorithm has shed new light on preexisting themes in gastric cancer etiology. The resulting bi-orderings represent successive steps in cancer progression and distinct histopathological types of the disease. Specifically, SBC1 represents epithelial morphology, typical to squamous samples; SBC2 and SBC8 are typical intestinal lipid metabolism signatures, observed in intestinal metaplasia pre-malignant samples; SBC3 and SBC4 represent a novel split of the inflammatory signature that in [1] were merged as one signature; SBC5 represents the proliferation signature described in [1] for intestinal type gastric cancer; SBC6 reflects the extracellular matrix deposition typical to diffuse type cancer, and elevated in all cancer samples compared to pre-malignant samples; SBC7 represents the metabolic stress observed in chronic gastritis samples, possibly due to elevated H. Pylori infection. There are also other observations which are potentially novel discoveries. They are available in the Additional file 1.

## 6 Conclusion

In this paper we have presented a novel method of bi-ordering genes and samples from microarray data, together with two statistical techniques for evaluating the significance of the generated groupings and orderings of multiple histological samples. In comparison to several existing algorithms in the literature, our method is able to generate highly robust and statistically significant gene modules with respect to sample histological annotations on a gastric cancer dataset. The results of our analysis closely match reported theories of gastric cancer tumorgenesis, and have helped to identify promising hypotheses for further investigation in cancer research. We also show that other biclustering algorithms can also be utilized as a basis of exploratory bi-ordering analysis of genomic data.

## Authors' contributions

Fan Shi, under the supervision of Christopher Leckie and Adam Kowalczyk, developed the major part of the algorithms. Geo MacIntyre contributed to the Gene Ontology evaluation of the results. Alex Boussioutas and Izhak Haviv analysed the biological relevance of the results. All authors contributed to the writing of the manuscript, and they have read and approved the final version of the manuscript.

## Supplementary Material

Additional file 1 **Supplement**. The Supplement contains the proof of convergence in a variant of BOA algorithm (See 2 for details), and the biological analysis of potential novel observations in the gastric cancer dataset discovered by our method. Click here for file

Additional file 2 **algorithm implementation**. The file of "algorithms.zip" contains the Matlab source code files (.m) implementing the BOA algorithm. Click here for file
