# Supplementary material for: A bi-ordering approach to linking gene expression with clinical annotations in gastric cancer
Source: BMC Bioinformatics. 2010 Sep 23;11:477. doi: 10.1186/1471-2105-11-477 (PMC2949898; doi:10.1186/1471-2105-11-477)
Supplement: Additional file 1 — Supplement. The Supplement contains the proof of convergence in a variant of BOA algorithm (See 2 for details), and the biological analysis of potential novel observations in the gastric cancer dataset discovered by our method. [file 1471-2105-11-477-S1.PDF]

A bi-ordering approach to linking gene expression with clinical annotations in gastric cancer
Additional File 1 – Supplement

Fan Shi*[1,2], Christopher Leckie[1,2], Geoff MacIntyre[1,2], Izhak Haviv[3] , Alex Boussioutas[4] , Adam Kowalczyk[1,2]

[1]National ICT Australia
[2]Department of Computer Science and Software Engineering,
[3]Baker IDI Heart and Diabetes Institute, 250 Kooyong Road Caulield, Victoria 3162, Australia
[4]Peter MacCallum Cancer Center, St Andrew's Place, East Melbourne, Victoria 3002, Australia

Email: Christopher Leckie - caleckie@csse.unimelb.edu.au; Geoff MacIntyre - gmaci@csse.unimelb.edu.au; Izhak Haviv - Izhak.Haviv@bakeridi.edu.au; Alex Boussioutas - alex.boussioutas@petermac.org; Adam Kowalczyk - a.kowalczyk@ee.unimelb.edu.au; Fan Shi*- shif@csse.unimelb.edu.au;

*Corresponding author

Comparison with Cheng & Church's Method

To make a systematic and fair comparison, we also applied Cheng and Church's method to the gastric cancer dataset that has been normalized by the procedure described in our BOA method. Subsequently, in order to compare the statistical significance of the results, the biclusters discovered by both Cheng and Church's method and our BOA method on the same normalized datasets were evaluated using the saturation metric (described in Section 2.3.1 in the paper) with respect to the pathological classification of samples. The p-values of both the single class and multiple classes saturation metric were plotted in Figure 1. The experimental settings of the figures are identical to Figure 2 in the paper. The figures demonstrate that the BOA method identified more significant biclusters than Cheng and Church's method with respect to the sample classes.

Convergence of Fixed-size Biclusters

The second variant of BOA (Section 2.1 in the paper) guarantees the convergence of biclusters, which is a desirable property for iterative algorithms. We formally prove that the algorithm converges if the subset of genes G and subset of samples S are selected with a fixed size at each iteration.

Theorem 1. *If the subsets of genes G and samples S are selected with fixed sizes (e.g., $|G| = \gamma$, $|S| = \sigma$) across all*

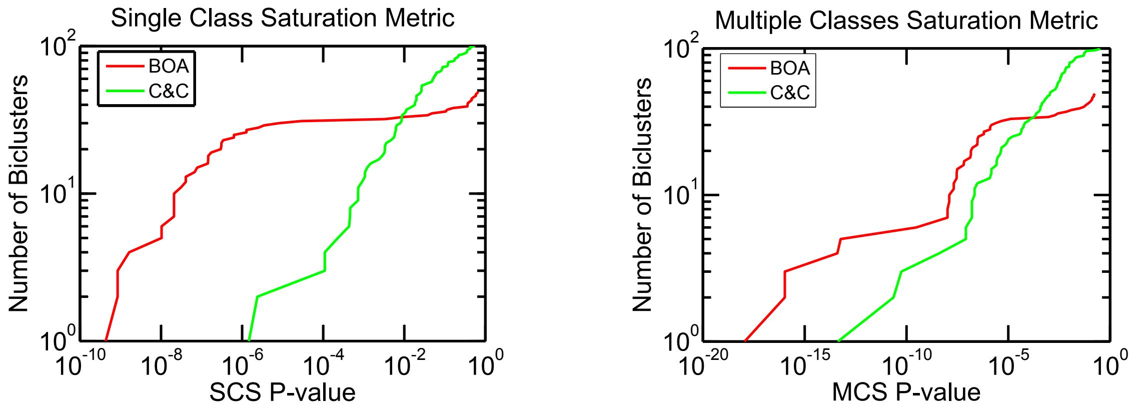

(a) Single Class Saturation (b) Multiple Classes Saturation

Figure 1: Saturation metric evaluation for BOA and Cheng and Church's method

iterations in the BOA algorithm (Section 2.1 in the paper), the algorithm is guaranteed to converge to a stationary bicluster (G and S do not change).

Proof. Let us introduce an objective function

$$F(G,S) := \sum_{g \in G} \sum_{s \in S} x_{gs},$$

defined for any subset of genes G and samples S.

We consider the following sequence of subsets of genes and samples, respectively, iteratively generated by the BOA algorithm at steps $j = 0, 1, 2, \cdots$:

$$S_0,\ G_0,\ S_1,\ \cdots,\ G_{i-1},\ S_i,\ G_i,\ S_{i+1},\ \cdots, \tag{1}$$

where S_0 is an initial subset of samples and S_i is selected based on G_{i-1} while G_i is selected based on S_i, etc.

In the particular case of fixed size subsets, the update of G_i or S_i is defined as follows:

$$G_i := \operatorname*{argmax}_{G, |G| = \gamma} F(G, S_i) \tag{2}$$

$$S_{i+1} := \operatorname*{argmax}_{S, |S| = \sigma} F(G_i, S). \tag{3}$$

After each step of refining genes or samples, we can re-calculate the objective function F_j for the current bicluster, i.e., the most recent values of G_i and $S_{i'}$:

$$F_j = \begin{cases} F(G_i, S_i), & \text{if } j = 2i,\ i = 0, 1, ...; \\ F(G_i, S_{i+1}), & \text{if } j = 2i + 1,\ i = 0, 1, ... \end{cases} \tag{4}$$

From (2) we find

$$F_{2i} = F(G_i, S_i) \le F(G_i, S_{i+1}) = F_{2i+1},$$

for $i = 0, 1, 2, \cdots$. Similarly, (3) yields

$$F_{2i+1} = F(G_i, S_{i+1}) \leq F(G_{i+1}, S_{i+1}) = F_{2i+2}.$$

Consequently, the objective function F_j is monotonically non-decreasing:

$$F_j \leq F_{j+1} \quad (5)$$

for $j = 0, 1, 2, \cdots$.

Moreover, we consider the condition that the equality holds in Equation (5). There are two possibilities for the equality. First, two consecutive sample subsets S_i and S_{i+1} are exactly the same, and we have $F_j = F_{j+1}$ (also for genes). In this case, the algorithm has converged since every selection based on the previous state is deterministic in BOA. Second, two consecutive sample (gene) subsets are not identical due to the draws in scores. To deal with this case, we employ a strategy as follows. If the objective function F_j does not improve in a certain number of iterations (usually 5 or 10), we could stop the algorithm. In this case, the subsets of genes or samples may be not stable due to the draws in the scores, but the objective functions stops improving. We can then select the current bicluster with the minimum F_j. In practice, the second case hardly ever occurs.

Except for the two special cases, we have the inequality $F_j < F_{j+1}$ (also for genes). In this case, a loop does not exist in the monotonically increasing sequence. Since there are only a finite number of states (biclusters), which are combinations of genes and samples, the sequence must converge to some state. Although the number of states (biclusters) is almost countless, it only affects the speed of convergence rather than the convergence itself. Moreover, the algorithm usually converges in less than 10 iterations in practice. □

Novel Discoveries

Of special interest are those observations from our analysis that highlight intriguing causality hypotheses. We now list those observations from Section 4.2 of the paper in detail.

1. Observation 1: Cancer cells are subject to both immune surveillance as well as boosting by the inflammatory response of the immune cells. These opposite effects are largely contributed by different cells, with the adaptive immunity largely attenuating cancer progression, and innate immunity promoting cancer. BOA has been able to split these two responses into SBC3 for innate immunity, and SBC4 for adaptive immunity.

2. Observation 2: SBC5 links the interaction of the epithelial cell with the extracellular matrix, with its level of proliferation. Specifically, a series of matrix metalloproteinases are elevated together with increased proliferation of the cancer cells.

3. Observation 3: SBC6, which clearly represents the worse prognostic value of cancer progression and further elevated in diffuse type cancers, has been described by our lab and others in the past ([Chang *et al.*, 2005]; [Segal *et al.*, 2004]). Here we observe for the first time, through the set of genes in SBC6, that a possible driver of this signature is elevation in the levels of a number of tyrosine kinase receptors known to be involved in tumorigenesis (Figure 2).

4. Observation 4: The lipid metabolism signature of intestinal cells, only observed in the stomach as a result of pathological processes such as intestinal metaplasia, relates to elevated lipid hormone signals, e.g., estrogen, testosterone and glucocortical receptor signaling.

Each of these four novel observations link to well known drug targets in cancer, i.e., non-steroidal anti-inflammatory drugs for Observation 1, matrix metalloproteinase inhibitors for Observation 2, tyrosine kinase inhibitors for Observation 3 and steroids in Observation 4. Consequently, these observations warrant further investigation in gastric cancer research, and highlight the value of our BOA algorithm for research on expression profiling of cancer.

References

Chang *et al.*, 2005. Chang,H.Y. *et al.* (2005) Robustness, scalability, and integration of a wound-response gene expression signature in predicting breast cancer survival, *Proc Natl Acad Sci U S A*.

Segal *et al.*, 2004. Segal,E. *et al.* (2004) A module map showing conditional activity of expression modules in cancer, *Nat Genet*, Vol. 36, No. 10, pp. 1090-8, 2004

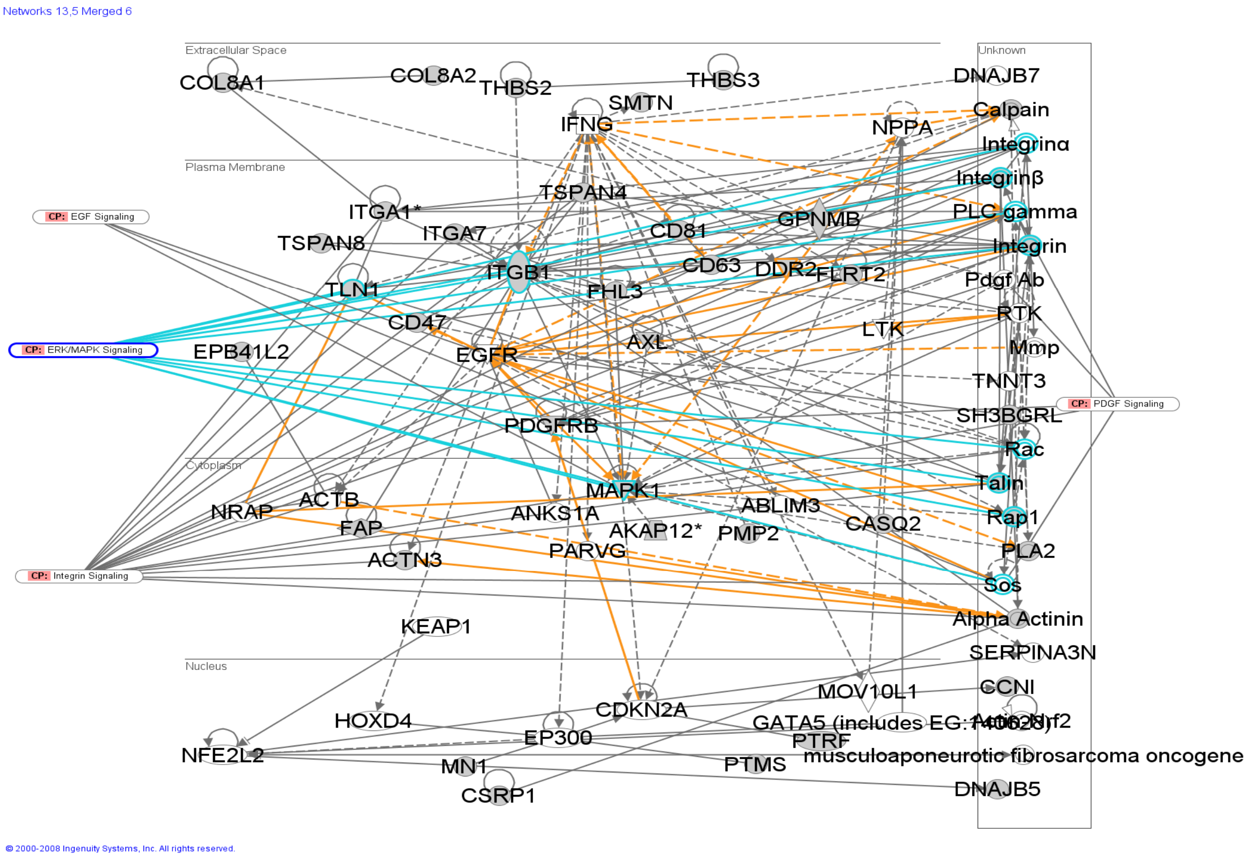

Figure 2: Signal transduction pathways predicted to operate in SBC6 signature-expressing cells (via analysis in Ingenuity Pathway Analysis®). Notice the genes PDGFRB, EGFR, DDR2, AXL and LTK are all tyrosine kinase receptors. Also noteworthy is ITGB1. The integrin and tyrosine kinase receptors both signal through the ERK pathway which is elevated in these samples.
